# The Dynamics of Attention Shifts Among Concurrent Speech in a Naturalistic Multi-speaker Virtual Environment

**DOI:** 10.3389/fnhum.2019.00386

**Published:** 2019-11-08

**Authors:** Keren Shavit-Cohen, Elana Zion Golumbic

**Affiliations:** The Gonda Multidisciplinary Brain Research Center, Bar Ilan University, Ramat Gan, Israel

**Keywords:** speech processing, auditory attention, eye-tracking, virtual reality, cocktail party effect, distractability

## Abstract

Focusing attention on one speaker on the background of other irrelevant speech can be a challenging feat. A longstanding question in attention research is whether and how frequently individuals shift their attention towards task-irrelevant speech, arguably leading to occasional detection of words in a so-called unattended message. However, this has been difficult to gauge empirically, particularly when participants attend to continuous natural speech, due to the lack of appropriate metrics for detecting shifts in internal attention. Here we introduce a new experimental platform for studying the dynamic deployment of attention among concurrent speakers, utilizing a unique combination of Virtual Reality (VR) and Eye-Tracking technology. We created a Virtual Café in which participants sit across from and attend to the narrative of a target speaker. We manipulated the number and location of distractor speakers by placing additional characters throughout the Virtual Café. By monitoring participant’s eye-gaze dynamics, we studied the patterns of overt attention-shifts among concurrent speakers as well as the consequences of these shifts on speech comprehension. Our results reveal important individual differences in the gaze-pattern displayed during selective attention to speech. While some participants stayed fixated on a target speaker throughout the entire experiment, approximately 30% of participants frequently shifted their gaze toward distractor speakers or other locations in the environment, regardless of the severity of audiovisual distraction. Critically, preforming frequent gaze-shifts negatively impacted the comprehension of target speech, and participants made more mistakes when looking away from the target speaker. We also found that gaze-shifts occurred primarily during gaps in the acoustic input, suggesting that momentary reductions in acoustic masking prompt attention-shifts between competing speakers, in line with “glimpsing” theories of processing speech in noise. These results open a new window into understanding the dynamics of attention as they wax and wane over time, and the different listening patterns employed for dealing with the influx of sensory input in multisensory environments. Moreover, the novel approach developed here for tracking the locus of momentary attention in a naturalistic virtual-reality environment holds high promise for extending the study of human behavior and cognition and bridging the gap between the laboratory and real-life.

## Introduction

Focusing attention on one speaker in a noisy environment can be challenging, particularly in the background of other irrelevant speech (McDermott, 2009). Despite the difficulty of this task, comprehension of an attended speaker is generally good and the content of distractor speech is rarely recalled explicitly (Cherry, 1953; Lachter et al., 2004). Preferential encoding of attended speech in multi-speaker contexts is also mirrored by enhanced neural responses to attended vs. distractor speech (Ding and Simon, 2012b; Mesgarani and Chang, 2012; Zion Golumbic et al., 2013b; O’Sullivan et al., 2015). However, there are also indications that distractor speech is processed, at least to some degree. Examples for this are the Irrelevant Stimulus Effect, where distractor words exert priming effect on an attended task (Treisman, 1964; Neely and LeCompte, 1999; Beaman et al., 2007), as well as occasional explicit detection of salient words in distractor streams (Cherry, 1953; Wood and Cowan, 1995; Röer et al., 2017; Parmentier et al., 2018). These effects highlight a key theoretical tension regarding how processing resources are allocated among competing speech inputs. Whereas Late-Selection models of attention posit that attended and distractor speech can be fully processed, allowing for explicit detection of words in so-called unattended speech (Deutsch and Deutsch, 1963; Duncan, 1980; Parmentier et al., 2018), Limited-Resources models hold that there are inherent bottlenecks for linguistic processing of concurrent speech due to limited resources (Broadbent, 1958; Lachter et al., 2004; Lavie et al., 2004; Raveh and Lavie, 2015). The latter perspective reconciles indications for occasional processing of distractor speech as stemming from rapid shifts of attention toward distractor speech (Conway et al., 2001; Escera et al., 2003; Lachter et al., 2004). Yet, despite the parsimonious appeal of this explanation, to date, there is little empirical evidence supporting and characterizing the psychological reality of attention switches among concurrent speakers.

Establishing whether and when rapid shifts of attention towards distractor stimuli occur is operationally challenging since it refers to individuals’ internal state that researchers do not have direct access to. Existing metrics for detecting shifts of attention among concurrent speech primarily rely on indirect measures such as prolongation of reaction times on an attended task (Beaman et al., 2007) or subjective reports (Wood and Cowan, 1995). Given these limitations, the current understanding of the dynamics of attention over time, and the nature and consequences of rapid attention-shifts among concurrent speech is extremely poor. Nonetheless, gaining insight into the dynamics of internal attention-shifts is critical for understanding how attention operates in naturalistic multi-speaker settings.

Here, we introduce a new experimental platform for studying the dynamic deployment of attention among concurrent speakers. We utilize Virtual Reality (VR) technology to simulate a naturalistic audio-visual multi-speaker environment, and track participant’s gaze-position within the Virtual Scene as a marker for the locus of overt attention and as a means for detecting attention-shifts among concurrent speakers. Participants experienced sitting in a “Virtual Café” across from a partner (avatar; animated target speaker) and were required to focus attention exclusively towards this speaker. Additional distracting speakers were placed at surrounding tables, with their number and location manipulated across conditions. Continuous tracking of gaze-location allowed us to characterize whether participants stayed focused on the target speaker as instructed or whether and how often they performed overt glimpses around the environment and toward distractor speakers. Critically, we tested whether shifting one’s gaze around the environment and away from the target speaker impacted comprehension of target speech. We further tested whether gaze-shifts are associated with salient acoustic changes in the environment, such as onsets in distractor speech that can potentially grab attention exogenously (Wood and Cowan, 1995) or brief pauses that create momentary unmasking of competing sounds (Lavie et al., 2004; Cooke, 2006).

Gaze-shifts are often used as a proxy for attention shifts in natural vision (Anderson et al., 2015; Schomaker et al., 2017; Walker et al., 2017), however this measure has not been utilized extensively in dynamic contexts (Marius’t Hart et al., 2009; Foulsham et al., 2011). This novel approach enabled us to characterize the nature of momentary attention-shifts in ecological multi-speaker listening conditions, as well as individual differences, gaining insight into the factors contributing to dynamic attention shifting and its consequences on speech comprehension.

## Materials and Methods

### Participants

Twenty-six adults participated in this study (ages 18–32, median 24; 18 female, three left handed), all fluent in Hebrew, with self-reported normal hearing and no history of psychiatric or neurological disorders. Signed informed consent was obtained from each participant prior to the experiment, in accordance with the guidelines of the Institutional Ethics Committee at Bar-Ilan University. Participants were paid for participation or received class credit.

### Apparatus

Participants were seated comfortably in an acoustic-shielded room and viewed a 3D VR scene of a café, through a head-mounted device (Oculus Rift Development Kit 2). The device was custom-fitted with an embedded eye-tracker (SMI, Teltow, Germany; 60 Hz monocular sampling rate) for continuous monitoring of participants’ eye-gaze position. Audio was presented through high-quality headphone (Sennheiser HD 280 pro).

### Stimuli

Avatar characters were selected from the Mixamo platform (Adobe Systems, San Jose, CA, USA). Soundtracks for the avatars’ speech were 35–50 s long segments of natural Hebrew speech taken from podcasts and short stories^1^. Avatars’ mouth and articulation movements were synced to the audio to create a realistic audio-visual experience of speech (LipSync Pro, Rogo Digital, England). Scene animation and experiment programming was controlled using an open-source VR engine (Unity Software^2^). Speech loudness levels (RMS) were equated for all stimuli, in 10-s long bins (to avoid biases due to fluctuations in speech time-course). Audio was further manipulated within Unity using a 3D sound algorithm, so that it was perceived as originating from the spatial location of the speaking avatar, with overall loudness decreasing logarithmically with distance from the listener. Participant’s head movements were not restricted, and both the graphic display and 3D sound were adapted on-line in accordance with head-position, maintaining a spatially-coherent audio-visual experience.

### Experiment Design

**Figure 1:**
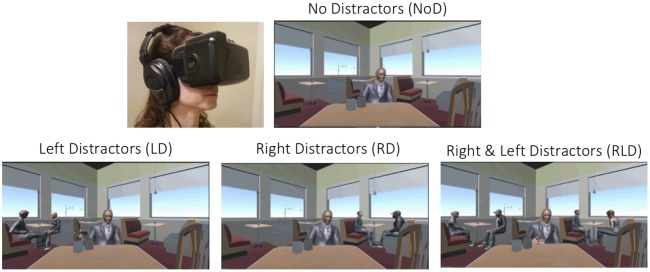
Manipulation of distraction in the Virtual Café. Participants are instructed to attend to the narrative of the target speaker facing them. The number and location of distractor speakers was manipulated in four conditions: only the target-speaker presented and No Distractors (NoD), a single distractor-pair sitting to the left (LD) or right (RD) of the target speaker, and two distractor-pairs sitting to the right and the left of the target speaker (RLD). Top-Left: demonstration of a participant experiencing the Virtual Café (written informed consent was obtained from the participant for publication of this photograph).

In the Virtual Café setting, participants experienced sitting at a café table facing a partner (animated speaking avatar) telling a personal narrative. They were told to focus attention exclusively on the speech of their partner (target speaker) and to subsequently answer four multiple-choice comprehension questions about the narrative (e.g., “What computer operating system was mentioned?”). Answers to the comprehension questions were evenly distributed throughout the narrative, and were pre-screened in a pilot study to ensure accuracy rates between 80% and 95% in a single-speaker condition. The time-period containing the answer to each question was recorded and used in subsequent analysis of performance as a function of gaze-shift behaviors (see below). Additional pairs of distracting speakers (avatars) were placed at surrounding tables, and we systematically manipulated the number and location of distractors in four conditions: No Distraction (NoD), Left Distractors (LD), Right Distractors (RD), Right and Left Distractors (RLD; Figure 1). Each condition consisted of five trials (~4 min per condition) and was presented in random order, which was different for each participant. The identity and voice of the main speaker were kept constant throughout the experiment, with different narratives in each trial, while the avatars and narratives serving as distractors varied from trial to trial. The allocation of each narrative to the condition was counter-balanced across participants, to avoid material-specific biases. Before starting the experiment itself, participants were given time to look around and familiarize themselves with the Café environment and the characters in it. During this familiarization stage, no audio was presented and participants terminated it when they were ready. They also completed two training-trials, in the NoD and RLD conditions, to familiarize them with the stimuli and task as well as the type of comprehension questions asked. This familiarization and training period lasted approximately 3-min.

### Analysis of Eye-Gaze Dynamics

Analysis of eye-gaze data was performed in Matlab (Mathworks, Natick, MA, USA) using functions from the fieldtrip toolbox^3^ as well as custom-written scripts. The position of eye-gaze position in virtual space coordinates (x, y, z) was monitored continuously throughout the experiment. Periods surrounding eye-blinks were removed from the data (250 ms around each blink). Clean data from each trial were analyzed as follows.

First, we mapped gaze-positions onto specific avatars/locations in the 3D virtual scene. For data reduction, we used a spatial clustering algorithm (k-means) to combine gaze data-points associated with similar locations in space. Next, each spatial cluster was associated with the closest avatar, by calculating the Euclidean distance between the center of the cluster and the center of each avatar presented in that condition. If two or more clusters were associated with looking at the same avatar, they were combined. Similarly, clusters associated with the members of the distractor avatar-pairs (left or right distractors) were combined. If a cluster did not fall within a particular distance-threshold from any of the avatars, it was associated with looking at “The Environment.” This resulted in a maximum of four clusters capturing the different possible gaze locations in each trial: (1) Target Speaker; (2) Left Distractors (when relevant); (3) Right Distractors (when relevant); and (4) Rest of the Environment. The appropriateness of cluster-to-avatar association and distance-threshold selection was verified through visual inspection.

Based on the clustered data, we quantified the percent of time that participants spent focusing at each location (*Percent Gaze Time*) in each trial, and detected the times of *Gaze-Shifts* from one cluster to another. Gaze-shifts lasting less than 250 ms were considered artifacts and removed from the analysis, as they are physiologically implausible (Bompas and Sumner, 2009; Gilchrist, 2011). The number of Gaze-shifts as well as the Percent Gaze Time spent at each of the four locations—Target Speaker, Left Distractors, Right Distractors and Environment—were averaged across trials, within condition. Since conditions differed in the type and number of distractors, comparison across conditions focused mainly on metrics pertaining to gazing at/away-from the target speaker.

Mixed linear regression models were used in all analyses to fit the data and test for effects of Condition on gaze patterns (both Percent Gaze-Time Away and Gaze-Shifts), as well as possible correlations with speech comprehension accuracy measures. These analyses were conducted in R (R Development Core Team, 2012) and we report statistical results derived using both regular linear (lme4 package for R; Bates et al., 2015) and robust estimation approaches (robustlmm package for R; Koller, 2016), to control for possible contamination by outliers. The advantage of mixed-effects models is that they account for variability between subjects and correlations within the data, as well as possible differences in trial numbers across conditions (Baayen et al., 2008), which makes them particularly suitable for the type of data collected here.

### Analysis of Speech Acoustics Relative to Gaze-Shifts

A key question is what prompts overt gaze-shifts away from the target speakers, and specifically whether they are driven by changes in the acoustic input or if they should be considered more internally-driven. Two acoustic factors that have been suggested as inviting attention-shifts among concurrent speech are: (a) onsets/loudness increases in distractor speech that can potentially grab attention exogenously (Wood and Cowan, 1995); and (b) brief pauses that create momentary unmasking of competing sounds (Lavie et al., 2004; Cooke, 2006). To test whether one or both of these factors account for the occurrence of gaze-shifts away from the target speaker in the current data, we performed a gaze-shift time-locked analysis of the speech-acoustics of target speech (in all conditions) and distractor speech (in the LD, RD and RLD conditions).

To this end, we first calculated the temporal envelope of the speech presented in each trial using a windowed RMS (30 ms smoothing). The envelopes were segmented relative to the times where gaze-shifts *away from the target speaker* occurred in that particular trial (−400 to +200 ms around each shift). Given that the initiation-time for executing saccades is ~200 ms (Gilchrist, 2011), the time-window of interest for looking at possible influences of the acoustics on gaze-shifts is prior to that, i.e., 400–200 ms prior to the gaze-shift itself.

Since the number of gaze-shifts varied substantially across participants, we averaged the gaze-shift-locked envelope-segments across all trials and participants, within condition. The resulting average acoustic-loudness waveform in each condition was compared to a distribution of non-gaze-locked loudness levels, generated through a permutation procedure as follows: the same acoustic envelopes were segmented randomly into an equal number of segments as the number of gaze-shifts in each condition (sampled across participants with the same proportion as the real data). These were averaged, producing a non-gaze-locked average waveform. This procedure was repeated 1,000 times and the real gaze-shift locked waveform was compared to the distribution of non-gaze-locked waveforms. We identified time-points where the loudness level fell above or below the top/bottom 5% tile of the non-gaze-locked distribution, signifying that the speech acoustics were particularly quiet or loud relative (relative to the rest of the presented speech stimuli). We also quantified the signal-to-noise ratio (SNR) between the time-resolved spectrograms of target and distractor speech surrounding gaze-shifts, according to: $SNR(f,t)=\log(\frac{P_{\text{target}}(f,t)}{P_{\text{distractor}}(f,t)})$, with *P(f,t)* depicting the power at frequency *f* at time *t*. This was calculated for target-distractor combinations surrounding each gaze-shift, and averaged across shifts and trials.

## Results

### Gaze-Patterns and Speech Comprehension

**Figure 2:**
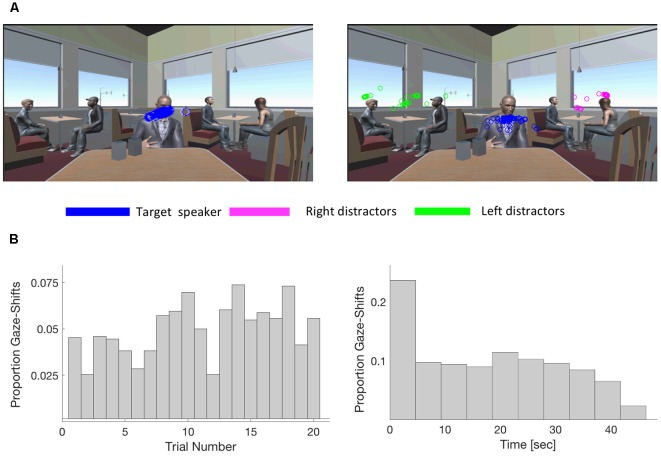
Characterization of gaze-shift patterns. **(A)** Illustration of the variability in gaze-patterns across individuals. The figure depicts all gaze data points in a specific trial in the RLD condition for two example participants. While the participant shown in the left panel remained focused exclusively on the target speaker throughout the trial (blue dots), the participant in the right panel spent a substantial portion of the trial looking at the distractor speakers on both the left (green) and the right (magenta). **(B)** Left: distribution of all gaze-shifts across the duration of the experiment, collapsed across participants. Gaze-shifts occurred throughout the experiment and were not more prevalent the beginning/end. Right: distribution of gaze-shifts over the course of a trial, collapsed across all trials and participants. Twenty-three percentage of gaze-shifts occurred during the first 5 s of each trial, and the remainder could occur with similar probability throughout the entire trial.

On an average, participants spent -7.6% of each trial (-3 s in a 40-s-long trial) looking at locations other than the target speaker and they performed an average of 2.5 gaze-shifts per trial. Figure 2A shows the distribution of eye-gaze location in two example trials taken from different participants, demonstrating that sometimes gaze was fixated on the target-speaker throughout the entire trial, and sometimes shifted occasionally towards the distractors. The distribution of Gaze-shifts was relatively uniform over the course of the entire experiment (Figure 2B, left). Twenty-three percentage of gaze-shifts were performed near the onset of the trial, however, the majority of gaze-shifts occurred uniformly throughout the entire trial (Figure 2B, right).

**Figure 3:**
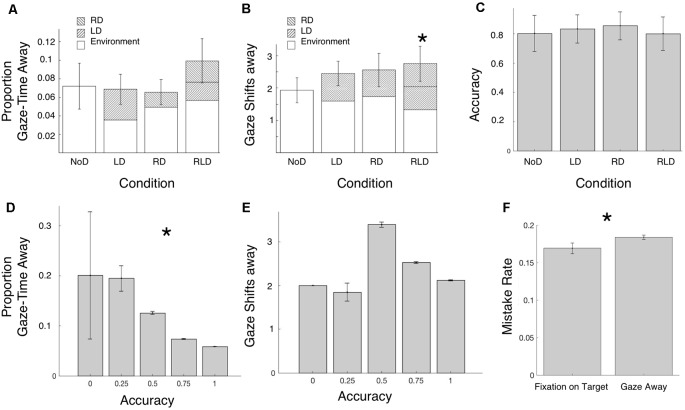
Summary of gaze-shift patterns and behavioral outcomes across conditions. **(A,B)** Proportion of Gaze-Time and Number of Gaze-Shifts Away from target speaker, per trial and across conditions. Results within each condition are broken down by gaze-location (Right Distractors, Left Distractors or Environment in blank, left and right diagonals, respectively). There was no significant difference between conditions in the total Gaze-time away from the target speaker or number of gaze-shifts. Significantly more Gaze-Shifts were performed in the RLD condition relative to the NoD condition. No other contrasts were significant. **(C)** Mean accuracy on comprehension questions, across condition. Difference between conditions was not significant. **(D,E)** Analysis of Accuracy as a function of Gaze-Shift Patterns, at the whole trial level. Trials where participants spent a larger proportion of the time looking away from the target-speaker were associated with lower accuracy rates. No significant correlation was found between accuracy rates and the number of Gaze-Shifts performed. **(F)** Analysis of Accuracy on single question as a function of Gaze-Shift Patterns. Mistake rates were significantly higher if participants were looking away from the target speaker vs. fixating on the target speaker during the time-window when the information critical for answering the question was delivered. Error bars indicate Standard Error of the Mean (SEM). *p < 0.05.

Figures 3A,B show how the average *Gaze Time* *Away* from the target speaker (i.e., time spent looking at distractor avatars or other locations in the Environment) and the number of *Gaze-Shifts* away from the target speaker, varied across the four conditions. To test whether gaze patterns (number of *Gaze-Shifts* and/or proportion *Gaze-Time* *Away*) differed across conditions, we estimated each of them separately using linear mixed effect model with the factor Condition as a fixed effect (Gaze-Shifts’ Condition and Gaze-Time–Condition), where each of the three distraction conditions (RD, LD and RLD) was compared to the NoD condition. By-subject intercepts were included as random effects. No significant effects of Condition were found on *Gaze-Time*, however, participants performed significantly more *Gaze-Shifts* in the RLD condition relative to the NoD condition (lmer: *β* = 0.8, *t* = 2.5, *p* = 0.01; robustlmm: *β* = 0.54, *t* = 2.5).

Of critical interest is whether the presence of distractors and gaze-shifts towards them impacted behavioral outcomes of speech comprehension. Accuracy on the multiple-choice comprehension questions of the target speaker was relatively good in all conditions (mean accuracy 82% ± 3; Figure 3C). A mixed linear model estimating Accuracy ~ Condition did not reveal any significant differences in Accuracy between conditions (lmer: all *t*’s < 0.199, *p* > 0.6; robustlmm: all *t*’s < 0.05). However, adding Percent Gaze-Time as a second fixed effect to the Accuracy ~ Condition model, improved the model significantly (*χ*^2^ = 9.14, *p* < 10^3^), with Percent Gaze-Time showing a significant correlation with Accuracy (lmer: *β* = −0.19, *t* = −3.13, *p* = 0.001; robustlmm: *β* = −0.23, *t* = −3.77; Figure 3D). Adding Number of Shifts to the Accuracy ~ Condition model, however, did not yield any additional significant advantage (likelihood ratio test *χ*^2^ = 2.4, *p* > 0.1; Figure 3E), suggesting that the number of gaze-shifts performed *per se* did not affect speech comprehension.

To further assess the link between performance on the comprehension questions and gaze-shifts, we tested whether participants were more likely to make mistakes on specific questions if they happened to be looking away from the target-speaker when the critical information for answering that question was delivered. Mistake rates were slightly lower when participants fixated on the target speaker when the critical information was delivered (16% miss-rate) vs. when they looked away (18% miss-rate). To evaluate this effect statistically, we fit a linear mixed model to the accuracy results on individual questions testing whether they were mediated by the presence of a gaze-shift when the answer was given, as well as the condition [Accuracy ~ Shift (yes/no) + Condition as fixed effects], with by-subject intercepts included as random effects. This analysis demonstrated a small yet significant effect of the presence of a gaze-shift during the period when the answer was given (lmer *β* = −0.05, *t* = −2.16, *p* < 0.04; robustlmm *t* = −3; Figure 3F), however there was no significant effect of Condition (all *t*’s < 0.5).

### Individual Differences in Gaze Patterns

**Figure 4:**
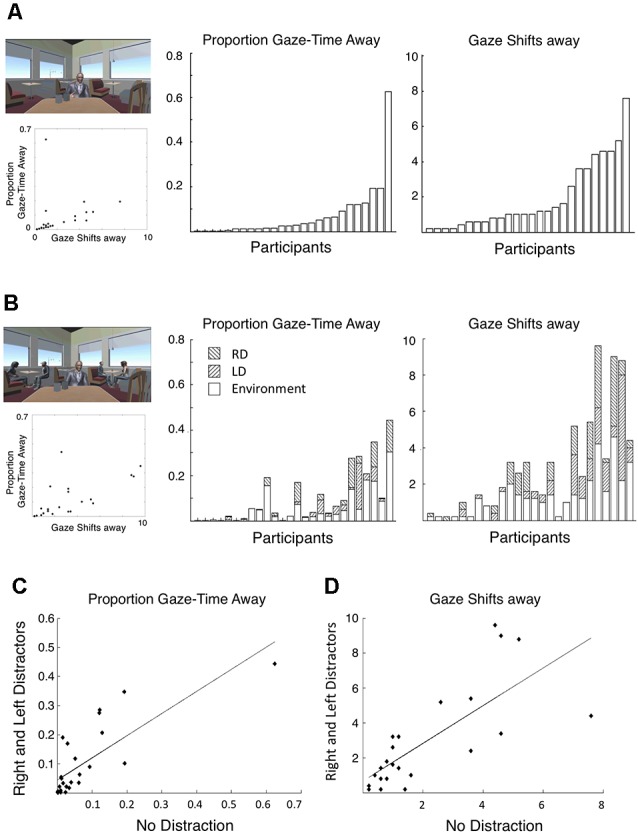
Individual gaze-shift patterns. **(A,B)** Proportion of time spent gazing away from the target speaker (left) and average number of gaze-shifts per trial (right) in the NoD condition **(A)** and the RLD conditions **(B)**, across individual participants. In both cases, participant order is sorted by the NoD condition (top panels). Scatter plots on the left indicate the relationship between the number of gaze-shifts and the proportion gaze-time away, across all participants in each condition. **(C,D)** Scatter plots depicting the relationship between the proportion of time spent gazing away from the target speaker **(C)** and average number of gaze-shifts per trial **(D)**, in the two extreme conditions: NoD vs. RLD. Correlations were significant in both cases (*r* > 0.5).

When looking at gaze-patterns across participants, we noted substantial variability in the number of gaze-shift performed and percent time spent gazing away from the target speaker. As illustrated in Figures 2A, 4, some participants stayed completely focused on the main speaker throughout the entire experiment, whereas others spent a substantial portion of each trial gazing around the environment (*range across participants*: 0–18 average number gaze-shifts per trial; 0–34.52% average percent of trial spent looking away from the target speaker). This motivated further inspection of gaze-shift behavior at the individual level. Specifically, we tested whether individual behavior of performing many or few gaze-shifts away from the target was stable across conditions. We calculated Cronbach’s α between conditions and found high internal consistency across conditions in the number of gaze-shifts performed as well as in the percent of gaze-time away from the target speaker (α = 0.889 and α = 0.832, respectively). This was further demonstrated by strong positive correlations between the percent time spent gazing away from the target speaker in No Distraction condition vs. each of the Distraction conditions (lmer: all *r*’s > 0.5; robustlmm all *r*’s > 0.6) as well as the number of gaze-shifts (lmer and robustlmm: all *r*’s > 0.5; Figures 4C,D). This pattern suggests that individuals have characteristic tendencies to either stay focused or gaze-around the scene, above and beyond the specific sensory attributes or degree of distraction in a particular scenario.

### Gaze-Locked Analysis of Speech Acoustics

**Figure 5:**
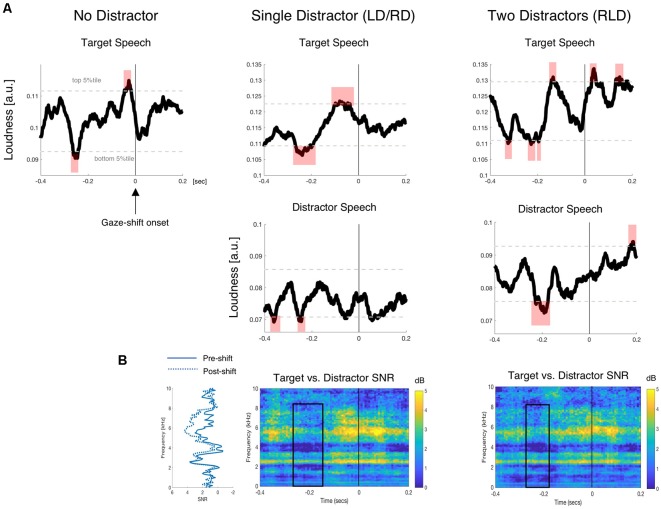
Gaze-shift locked analysis of speech acoustics. **(A)** Average time-course of the target (top) and distractor (bottom) speech envelopes relative to gaze shift onset (*t* = 0). Horizontal dotted gray lines depict the top and bottom 5%tile of loudness values generated through the permutation procedure of non-gaze-locked acoustics segments. The shaded red areas indicate time-periods where the speech sound-level fell within the lower/upper 5% tile of the distribution, respectively. **(B)** Spectrograms depicting the signal-to-noise ratio (SNR) between the target and distractor speaker(s), surrounding the onset of a gaze-shift, in the single and two-distractor conditions. A reduction in SNR is seen in a 200 ms pre-shift time window, primarily in the higher “unvoiced” portion of the spectrogram (4–8 KHz).

Last, we tested whether there was any relationship between the timing of gaze-shifts and the local speech-acoustics. To this end, we performed a gaze-shift-locked analysis of the envelope of the target or distractor speech (when present). Analysis of distractor speech envelope consisted only of eye-gaze shifts *toward* *that* *distractor* (i.e., excluding shifts to other places in the environment). Figure 5 shows the average time-course of the target and distractor speech envelopes relative to the onset of a gaze-shift. For both target speech (top row) as well as for distractor speech (bottom row), gaze-shifts seem to have been preceded by a brief period of silence (within the lower 5% tile; red shading) between 200 and 300 ms prior to the shift.

Frequency-resolved analysis of the SNR between target and distractor speech similarly indicates low SNR in the period preceding gaze-shifts. A reduction in SNR prior to gaze-shifts was primarily evident in the 3–8 kHz range (sometimes considered the “unvoiced” part of the speech spectrum; Atal and Hanauer, 1971), whereas SNR in the lower part of the spectrum (0–2 kHz) was near 1 dB both before and after gaze-shifts. Although SNR does not take into account the overall loudness-level of each speaker but only the ratio between the speakers, the observed SNR modulation is consistent with momentary periods of silence/drops in the volume of both concurrent speakers.

This pattern is in line with an acoustic release-from-masking account, suggesting that gaze-shifts are prompted by momentary gaps in the speech, and particularly when gaps in concurrent speech coincide-temporally (as seen here in the Single and Two Distractor conditions). Conversely, the suggestion that attention-shifts are a product of exogenous capture by salient events in distracting speech does not seem to be supported by the current data, since the acoustics of the distractor speech that participants shifted their gaze towards did not seem to contain periods with consistently loud acoustics. We did, however, find increases in loudness of the target speech acoustics near gaze-shift onset (within the top 5% tile; red shading between −100 and +50 ms).

## Discussion

The current study is a first and novel attempt to characterize how individuals deploy overt attention in naturalistic audiovisual settings, laden with rich and competing stimuli. By monitoring eye-gaze dynamics in our Virtual Café, we studied the patterns of gaze-shifts and its consequences for speech comprehension. Interestingly, we found that the presence and number of competing speakers in the environment did not, on average, affect the amount of time spent looking at the target speaker, nor did it impair comprehension of the target speaker, although participants did perform slightly more gaze-shifts away in the two-distractor RLD condition. This demonstrates an overall resilience of the attention and speech-processing systems for overcoming the acoustic-load posed by distractors in naturalistic audio-visual conditions. This ability is of utmost ecological value, and likely benefits both from the availability of visual and spatial cues (Freyman et al., 2004; Zion Golumbic et al., 2013a) as well as the use of semantic context to maintain comprehension despite possible reductions in speech intelligibility (Simpson and Cooke, 2005; Vergauwe et al., 2010; Ding and Simon, 2012a; Calandruccio et al., 2018). At the same time, our results also suggest that the ability to maintain attention on the designated speaker under these conditions is highly individualized. Participants displayed characteristic patterns of either staying focused on a target speaker or sampling other locations in the environment overtly, regardless of the severity of the so-called sensory distraction. Critically, the amount of time that individuals spent looking around the environment and away from the target speaker was negatively correlated with speech comprehension, directly linking overt attention to speech comprehension. We also found that gaze-shifts away from the target speaker occurred primarily following gaps in the acoustic input, suggesting that momentary reductions in acoustic masking can prompt attention-shifts between competing speakers, in line with “glimpsing” theories of processing speech in noise. These results open a new window into understanding the dynamics of attention as they wax and wane over time, and the listening patterns exhibited by individuals for dealing with the influx of sensory input in complex naturalistic environments.

### Is Attention Stationary?

An underlying assumption of many experimental studies is that participants allocate attention solely to task-relevant stimuli, and that attention remains stationary over time. However, this assumption is probably unwarranted (Weissman et al., 2006; Esterman et al., 2013) since sustaining attention over long periods of time is extremely taxing (Schweizer and Moosbrugger, 2004; Warm et al., 2008; Avisar and Shalev, 2011), and individuals spend a large proportion of the time mind-wandering or “off-task” (Killingsworth and Gilbert, 2010; Boudewyn and Carter, 2018; but see Seli et al., 2018). Yet, empirically testing the studying the frequency and characteristics of attention shifts is operationally difficult since it pertains to participants’ internal state that experimenters do not have direct access to. The use of eye-gaze position as a continuous metric for the locus of momentary overt attention in a dynamic scene in the current study contributes to this endeavor.

Here, we found that indeed, in many participants eye-gaze was not maintained on the target speaker throughout the entire trial. Roughly 30% of participants spent over 10% of each trial looking at places in the environment other than the to-be-attended speaker, across all conditions. Interestingly, this proportion is similar to that reported in previous studies for the prevalence of detecting ones’ own name in a so-called unattended message (Cherry, 1953; Wood and Cowan, 1995), an effect attributed by some to rapid attention shifts (Lachter et al., 2004; Beaman et al., 2007; Lin and Yeh, 2014). Although in the current study we did not test whether these participants also gleaned more information from distractors’ speech, we did find that comprehension of the target speaker was reduced as a function of the time spent looking away from the target speaker. Participants were also more likely to miss information from the target-speech during gaze-shifts away, yielding slightly higher mistake-rates. These results emphasize the dynamic nature of attention and attention-shifts, and demonstrate that brief overt attention-shifts can negatively impact speech processing in ecological multi-speaker and multisensory contexts.

They also highlight the importance of studying individual differences in attentional control. In the current study set, we did not collect additional personal data from participants which may have shed light on the source of the observed variability in gaze-patterns across individuals. However, based on previous literature, individual differences may stem from factors such as susceptibility to distraction (Ellermeier and Zimmer, 1997; Cowan et al., 2005; Avisar and Shalev, 2011; Bourel-Ponchel et al., 2011; Forster and Lavie, 2014; Hughes, 2014), working memory capacity (Conway et al., 2001; Kane and Engle, 2002; Tsuchida et al., 2012; Sörqvist et al., 2013; Hughes, 2014; Naveh-Benjamin et al., 2014; Wiemers and Redick, 2018) or personality traits (Rauthmann et al., 2012; Risko et al., 2012; Baranes et al., 2015; Hoppe et al., 2018). Additional dedicated research is needed to resolve the source of the individual differences observed here.

### Is Eye-Gaze a Good Measure for Attention-Shifts Among Concurrent Speech?

One may ask, to what extent do the current results fully capture the prevalence of attention-shifts, since it is known that these can also occur covertly (Posner, 1980; Petersen and Posner, 2012)? This is a valid concern and indeed the current results should be taken as representing a *lower-bound* for the frequency of attention-shifts and we should assume that attention-shifts are probably more prevalent than observed here. This motivates the future development of complementary methods for quantifying covert shifts of attention among concurrent speech, given the current absence of a reliable metrics.

Another concern that may be raised with regard to the current results is that individuals may maintain attention to the target speaker even while looking elsewhere, and hence the gaze-shifts measured here might not reflect true shifts of attention. Although in principle this could be possible, previous research shows that this is probably not the default mode of listening under natural audiovisual conditions. Rather, a wealth of studies demonstrate a tight link between gaze-shifts and attention-shifts (Chelazzi et al., 1995; Deubel and Schneider, 1996; Grosbras et al., 2005; Szinte et al., 2018) and gaze is widely utilized experimentally as a proxy for the locus of visuospatial attention (Gredebäck et al., 2009; Linse et al., 2017). In multi-speaker contexts, it has been shown that participants tend to move their eyes towards the location of attended speech sounds (Gopher and Kahneman, 1971; Gopher, 1973). Similarly, looking towards the location of distractor-speech significantly reduces intelligibility and memory for attended speech and increases intrusions from distractor speech (Reisberg et al., 1981; Spence et al., 2000; Yi et al., 2013). This is in line with the current finding of a negative correlation between the time spent looking at the target speaker and speech comprehension, and higher mistake-rates during gaze-shifts, which further link overt gaze to selective attention to speech. Studies on audiovisual speech processing further indicate that looking at the talking face increases speech intelligibility and neural selectivity for attended speech (Sumby and Pollack, 1954; Zion Golumbic et al., 2013a; Lou et al., 2014; Crosse et al., 2016; Park et al., 2016), even when the video is not informative about the content of speech (Kim and Davis, 2003; Schwartz et al., 2004), and eye-gaze is particularly utilized for focusing attention to speech under adverse listening condition (Yi et al., 2013). Taken together, current findings support the interpretation that gaze-shifts reflect shifts in attention away from the target speaker, in line with the limited resources perspective of attention (Lavie et al., 2004; Esterman et al., 2014), making eye-gaze a useful and reliable metric for studying the dynamics of attention to naturalistic audio-visual speech. Interestingly, this metric has recently been capitalized on for use in assistive listening devices, utilizing eye-gaze direction to indicate the direction of a listener’s attention (Favre-Felix et al., 2017; Kidd, 2017). That said, gaze-position is likely only one of several factors in determining successful speech comprehension in multi-speaker environments (e.g., SNR level, audio-visual congruency, engagement in content etc.), as suggested by the significant yet still moderate effect-sizes found here.

### Listening Between the Gaps—What Prompts Attention Shifts Among Concurrent Speech?

Besides characterizing the prevalence and behavioral consequences of attention-shifts in audio-visual multi-talker contexts, it is also critical to understand what prompts these shifts. Here we tested whether there are aspects of the scene acoustics that can be associated with attention-shifts away from the target speaker. We specifically tested two hypotheses: (1) that attention is captured exogenously by highly salient sensory events in distracting speech (Wood and Cowan, 1995; Itti and Koch, 2000; Kayser et al., 2005); and (2) that attention-shifts occur during brief pauses in speech acoustics that momentarily unmask the competing sounds (Lavie et al., 2004; Cooke, 2006).

Regarding the first hypothesis, the current data suggest that distractor saliency is not a primary factor in prompting gaze-shifts. Since gaze-shifts were just as prevalent in the NoD condition as in conditions that contained distractors and since no consistent increase in distractor loudness was observed near gaze-shifts, we conclude that the gaze-shifts performed by participants do not necessarily reflect exogenous attentional capture by distractor saliency. This is in line with previous studies suggesting that sensory saliency is less effective in drawing exogenous attention in dynamic scenarios relative to the stationary contexts typically used in laboratory experiments (Smith et al., 2013).

Rather, our current results seem to support the latter hypothesis that attention-shifts are prompted by momentary acoustic release-from-masking. We find that gaze-shifts occurred more consistently ~200–250 ms after instances of low acoustic intensity in both target and distractor sounds and low SNR. This time-scale is on-par with the initiation time for saccades (Gilchrist, 2011), and suggests that momentary reduction in masking provide an opportunity for the system to shift attention between speakers. This pattern fits with accounts for comprehension of speech-in-noise, suggesting that listeners utilize brief periods of unmasking or low SNR to glean and piece together information for deciphering speech content (“acoustic glimpsing”; Cooke, 2006; Li and Loizou, 2007; Vestergaard et al., 2011; Rosen et al., 2013). Although this acoustic-glimpsing framework is often used to describe how listeners maintain intelligibility of target-speech in noise, it has not been extensively applied to studying *shifts* of attention among concurrent speech. The current results suggest that brief gaps in the audio or periods of low SNR may serve as triggers for momentary attention shifts, which can manifest overtly (as demonstrated here), and perhaps also covertly. Interestingly, a previous study found that eye-blinks also tend to occur more often around pauses when viewing and listening to audio-visual speech (Nakano and Kitazawa, 2010), pointing to a possible link between acoustic glimpsing and a reset in the oculomotor system, creating optimal conditions for momentary attention-shifts.

## Conclusion

There is growing understanding that in order to really understand the human cognitive system, it needs to be studied in contexts relevant for real-life behavior, and that tightly constrained artificial laboratory paradigms do not always generalize to real-life (Kingstone et al., 2008; Marius’t Hart et al., 2009; Foulsham et al., 2011; Risko et al., 2016; Rochais et al., 2017; Hoppe et al., 2018). The current study represents the attempt to bridge this gap between the laboratory and real-life, by studying how individuals spontaneously deploy overt attention in a naturalistic virtual-reality environment. Using this approach, the current study highlights the characteristics and individual differences in selective attention to speech under naturalistic listening conditions. This pioneering work opens up new horizons for studying how attention operates in real-life and understanding the factors contributing to success as well as the difficulties in paying attention to speech in noisy environments.

## Data Availability Statement

The datasets generated for this study are available on request to the corresponding author.

## Ethics Statement

The study was approved by the Institutional Ethics Committee at Bar-Ilan University, and the research was conducted according to the guidelines of the committee. Signed informed consent was obtained from each participant prior to the experiment.

## Author Contributions

EZG designed the study, oversaw data collection and analysis. KS-C collected and analyzed the data. Both authors wrote the article.

## Conflict of Interest

The authors declare that the research was conducted in the absence of any commercial or financial relationships that could be construed as a potential conflict of interest.

## Footnotes

^1^www.icast.co.il

^2^unity3d.com

^3^fieldtriptoolbox.org
